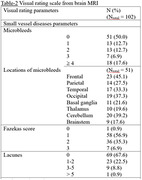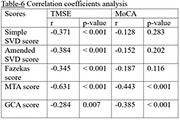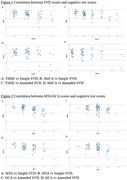# Prevalence of Cerebral Small Vessel Lesions in Mild Cognitive Impairment and Mild Alzheimer’s Disease Patients in Memory Clinic

**DOI:** 10.1002/alz.088254

**Published:** 2025-01-09

**Authors:** Thachamai Smitasiri, Yuttachai Likitjaroen

**Affiliations:** ^1^ Chulalongkorn University, Bangkok, Bangkok Thailand; ^2^ Neurocognitive Unit, Division of Neurology, Faculty of Medicine, Chulalongkorn University, Bangkok Thailand

## Abstract

**Background:**

Alzheimer's disease(AD) raises global concern with its impact on daily living. Anti‐amyloid monoclonal antibodies (mAb) serving as specific treatments used in mild cognitive impairment(MCI) and mild dementia due to AD. Severe cerebral small vessel disease(SVD) lesions such as microbleeds and white matter hyperintensities are listed as exclusions according to the recommendation for mAb treatment. In Thailand, there is no prevalence study of SVD lesions specifically in these patient grpups. The findings will provide important information for considering cost‐effectiveness of anti‐amyloid therapy, being expected to become treatment option for Thai AD patients.

**Method:**

The cross‐sectional study was carried out at King Chulalongkorn Memorial Hospital Memory Clinic. Amnestic MCI and mild AD patients were diagnosed by clinical criteria, cognitive tests and brain MRI. Serious medical conditions and incomplete MRI were excluded. Visual rating scales were used to evaluate SVD and brain atrophy lesions (microbleeds, Fazekas score, lacunes, MTA and GCA scores). Baseline characteristics data and cognitive tests were accessed from medical records. Simple and amended SVD scores were used to determine SVD severity and analyzed correlation with cognitive tests.

**Result:**

In the study involving 102 patients, 73.5% had MCI, and 26.5% had mild AD, with average age at diagnosis of 75.0±8.0 years. Microbleeds were present in 50% of patients, with only 17.6% had ³4 lesions. Twenty individuals (19.6%) had either ³4 microbleed lesions or Fazekas score of 3. There was no evidence of superficial siderosis or stroke involving large vessel territory. Those without microbleeds had higher TMSE score (25.4vs23.5;p=0.009). MTA score were associated with lower TMSE and MoCA scores (26.4vs22.7;p<0.001 and 22.1vs19.2;p=0.001, respectively). Weak negative correlation between simple & amended SVD scores and TMSE scores were showed (r=‐0.371 and ‐0.384, respectively;p<0.001), along with moderate negative correlation between MTA score and both TMSE & MoCA score (r=‐0.631 and ‐0.443, respectively;p<0.001).

**Conclusion:**

With MCI and mild AD, 19.6% of the samples fell within the exclusion criteria for anti‐amyloid therapy, in part of cerebrovascular imaging lesions. The results can be used to consider role of anti‐amyloid therapy in Thailand in the future.